# ‘Phasing out pig tail docking in the EU - present state, challenges and possibilities’

**DOI:** 10.1186/s40813-018-0103-8

**Published:** 2018-11-16

**Authors:** Nancy De Briyne, Charlotte Berg, Thomas Blaha, Andreas Palzer, Déborah Temple

**Affiliations:** 1 Federation of Veterinarians of Europe, Avenue Tervueren 12, 1040 Brussels, Belgium; 2 Department of Animal Environment and Health, Swedish University of Agricultural Sciences, POB 234, SE-532 23 Skara, Sweden; 3 German Veterinary Association for Animal Welfare, Wiesenweg 11, 49456 Bakum, Germany; 4 Clinic for Swine, Ludwig-Maximilians-University Munich, Sonnenstrasse 16, 85764 Oberschleissheim, Germany; 5 Universitat Autònoma de Barcelona, Farm Animal Welfare Education Center, Plaza Cívica, s/n, 08193 Bellaterra, Barcelona Spain

**Keywords:** Animal welfare, Enrichment materials, Mutilations, Straw, Swine, Tail biting, Veterinarian

## Abstract

**Background:**

European legislation dictates that pig tail docking is not allowed to be performed routinely (European Union. Council Directive 2008/120/EC of 18 December 2008 laying down minimum standards for the protection of pigs. OJ L 47, 18.2.2009). Nevertheless, tail docking is still practiced routinely in many European countries, while four countries stopped routine tail docking completely. Tail docking is also practiced in many countries outside Europe.

The Federation of Veterinarians of Europe (FVE), the European Association of Porcine Health Management (EAPHM) together with the European Commission carried out an online survey to investigate the situation regarding the practice of pig tail docking and the provision of enrichment material across 24 European countries. It also focuses on the role of the veterinary profession and gives an overview on published literature regarding the challenges and possibilities related to the raising of pigs with intact tails.

**Results:**

Fifty-seven (57) usable survey responses from 24 countries were received. On average 77% (median = 95%) of pigs are routinely tail-docked. In Finland, Norway, Sweden, Switzerland, less than 5% of the pigs are tail-docked. According to the respondents, 67% of pigs (median = 76%) across the 24 EU countries surveyed are given suitable enrichment materials. Training of veterinary practitioners, their role in advising the producer and undertaking a risk assessment of tail biting were more positively valued in countries that stopped routine tail docking than in countries that had not stopped routine tail docking. Initiatives such as training from national authorities to encourage abandoning tail docking and routine recording of tail biting at the slaughterhouse were identified as two successful items to promote the raising of pigs with entire tails.

**Conclusion:**

In many European countries the majority of the pigs are still routinely tail-docked, which is a violation of the European legislation. To stop routine tail docking it is necessary to raise the awareness and education about risk factors to prevent tail biting. The growing knowledge about the reasons for failing voluntary national initiatives as well as about successful measures taken by some countries to make pig production with intact tails feasible should be distributed throughout the EU pig producing community. The veterinary profession has a significant role to play in raising awareness, facilitate knowledge transfer and to identify risk factors and solutions on farm level for the benefit of pig health and welfare.

## Background

Many piglets in Europe are routinely exposed to the cutting or cauterizing of a part of the tail in the first week after birth to prevent tail biting in their later life. This procedure is mostly done by the farmer during the first week of the piglets’ life, without any pain relief. If it is done after the piglets are 7 days old, it needs to be performed by a veterinarian with provision of analgesia/anaesthesia to provide pain relief, in accordance with the EU pig Directive [1].

Since 1994, European legislation has stipulated that this procedure cannot be done routinely and only if there is evidence of tail biting and if other measures have first been taken to prevent tail biting but have failed. Suggested measures to be taken include improving husbandry and environmental conditions, increasing the space allowance, modifying the management system or providing additional enrichment material.

The European Food Safety Authority (EFSA) 2007 scientific opinion [2] on risk factors for tail biting in pigs reported that the practice of tail docking was widespread in the vast majority of EU Member States, with percentages of 81–100% of pigs being tail docked, the only exceptions being Finland (5% of pigs tail-docked), Lithuania and Sweden (0%). Three EU Member States - Austria, Denmark and Slovenia - have specific legislation further limiting this practice, while 3 others - Finland, Lithuania and Sweden – have prohibited tail docking, unless motivated in each individual case from a veterinary perspective.

Tail biting is associated with pain, stress and frustration and negatively affects food safety [2]. It has the potential for evoking short- as well as long-term physiological and behavioural changes indicative of pain [3, 4]. It can be triggered by a wide range of factors, often in combination, including: overstocking, feed and drinking water deficiencies or competition for these resources, incorrect or fluctuating temperature levels, inadequate ventilation, noise, draught, high levels of dust and noxious gases (i.e. ammonia), lack of opportunities to escape dominant animals, genetic factors, lack of environmental enrichment such as rooting material, and also general health problems [2, 5, 6]. If tail biting occurs, it can spread quickly through affected and neighbouring pens, can be difficult to stop and the degree of injury can increase rapidly [5, 7]. Tail biting can occur in all production systems. Danish studies which compared slaughter lesions in pigs found more tail lesions in free-range systems than in the conventional indoor system. Marked herd effects were noted [8, 9].

Often tail biting prevention is part of the farm animal health and welfare plan, which the farmer prepares together with the contracted veterinarian. Together they have to ensure that enough and appropriate enrichment material is provided and that husbandry, management and climate conditions are optimal [10, 11]. In some countries, if all these preventive steps are taken and the farmer still needs to tail-dock, the veterinarian has to sign a veterinary certificate to verify this and to justify the pigs being tail-docked. Trade issues also play a role, with some fattening farms only wishing to purchase tail-docked piglets.

Tail biting is a significant animal health, welfare and food safety problem. As a result of increased numbers of dead and runted animals after ascending abscesses it can also pose a considerable financial problem to the farmer, and increased costs for carcass handling at the slaughterhouse. An Irish study with tail-docked pigs calculated producer losses resulting from carcass condemnation and carcass trimmings to be around €1.1 per pig slaughtered [12]. These combined losses represented a loss of 43% of the profit margin per pig, at the time of the study, attributable to tail biting. ProHealth, an FP7 Framework program, estimated the costs of tail biting in tail-docked fattening pigs at about €2 per produced pig [13]. Tail docking is used to reduce the risk of tail biting. Nevertheless, tail docking is in itself a welfare problem, as it causes pain to the pigs, can lead to the formation of spinal abscesses, impairs the physical integrity of the animals, and facilitates suboptimal production methods from a welfare point-of-view and it does not completely remove the risk for tail biting [14]. When evaluating the costs and benefits of tail docking, it is important to consider negative impacts of both tail docking and tail biting [14].

In addition to the ban on routine tail docking, European legislation requires that pigs must have “*permanent access to a sufficient quantity of material to enable proper investigation and manipulation activities, such as straw, hay, wood, sawdust, mushroom compost, peat or a mixture of such*” [1]. In order to give more clarity on measures to prevent the need for tail docking and on ‘suitable enrichment’, in 2016 the European Commission adopted recommendations on tail biting and suitable enrichment materials [15, 16]. Many scientific publications also emphasized the importance of providing suitable enrichment material and the relation of it with tail biting [2, 14, 17, 18].

In the last years a high level of political attention has been seen to improve implementation and enforcement of the ban on routine tail docking [19–21].

The aim of this paper was to analyse the progress made in the different countries regarding phasing out of tail docking. Specific attention was given to the role of veterinary practitioners in the efforts to reduce tail biting and docking.

## Methodology

This publication is based on an online survey, interviews with regional pig experts and an investigation of (scientific) opinions on pig tail biting, tail docking and provision of enrichment materials. The online survey on pig tail docking was designed by FVE, EAPHM and the European Commission, Directorate General for Health and Food Safety via SurveyMonkey©. It was distributed to all national veterinary organisations (specifically requesting to forward them and let them be filled in by pig experts) and to members of the EAPHM, the European College of Porcine Health Management (ECPHM) and pig experts from the European College of Animal Welfare and Behavioural Medicine (ECAWBM) between 10 April 2017 and 30 October 2017. In total, 60 surveys from 24 countries were received and 57 of them provided usable answers. The final number of respondents per country varied from 1 to 7. Each respondent was asked about the estimated percentage of i) tail-docked pigs; ii) provision of suitable enrichment materials. The survey also contained questions regarding the risk factors, challenges, slaughterhouse monitoring and role of the veterinary practitioner. All questions are listed in Appendix.

### Statistics

The tail docking outcome was converted into dichotomous variable to look for possible associations between variables. Each respondent was classified in one of two categories based on expert opinion on the percentages provided by the survey: 0) No-TD (No-Tail Docking): no or few tail-docked pigs (range from 0 to 5%) and 1) TD (Tail Docking): more or equal than 70% of tail-docked pigs (range from 70 to 100%). Responses from the 3 countries reporting intermediate tail docking levels were not included in this part of the analysis.

The experimental unit was the respondent. A Mann–Whitney Wilcoxon test was used to identify any significant difference between the prevalence of suitable enrichment material in countries which routinely tail-dock versus those that phased out tail docking. A chi-square test was applied to detect possible associations between the other answers given and the fact of performing routine tail docking or not. A *p*-value of 0.05 was considered significant for all analyses.

## Results

### Percentages of pigs’ tail-docked

Table 1 shows the percentage of pigs’ tail-docked and given enrichment materials in the 24 countries that participated in the survey. On average 77% (median = 95%) of pigs were routinely tail-docked. In Finland, Norway, Sweden, Switzerland, less than 5% of the pigs were tail-docked. Respondents from Estonia, Malta and Serbia reported intermediate percentages (> 5% to < 70%) of tail-docked pigs.

**Table 1:** Percentage of pigs tail docked and pigs provided suitable enrichment material (mean, max and min) in relation to the pig population in the 24 countries surveyed

Country (number of usable answers)	Tail-docked	Enrichment material	Pig population^a^
	Mean % (range)	Mean % (range)	
Austria (2)	92.5 (90–95)	60 (30–90)	2792
Belgium (7)	97 (95–100)	78 (10–100)	6176
Czech (1)	90	60	1479
Denmark (1)	98	97	12,281
Estonia (1)	45	90	266
Finland (2)	1.5 (0–3)	85 (72–98)	1197
France (5)	95 (85–99)	72 (10–99)	12,793
Germany (3)	89 (80–99)	95 (90–99)	27,376
Hungary (1)	70	40	2907
Italy (4)	94.5 (90–100)	44 (30–70)	8477
Ireland (2)	97.5 (96–99)	46 (16–76)	1527
Latvia (1)	90	10	336
Luxembourg (1)	95	95	95
Malta (1)	56	45	41
Netherlands (5)	91.8 (88–97)	52.4 (25–100)	11,881
Norway (1)	0	60	1644
Poland (2)	95	55 (20–90)	11,107
Romania (2)	100 (100)	87.5 (75–100)	4707
Serbia (1)	60	60	3200
Slovakia (1)	98	20	585
Spain (5)	94.6 (90–98)	39.4 (5–100)	29,231
Sweden (4)	0	97,25 (90–99)	1471
Switzerland (2)	2.5 (0–5)	90 (80–100)	1442
UK (2)	84 (70–98)	91.75 (89–100)	4538
Europe-24 total pig population	77% (median = 95%)	67% (median = 76%)	147,549

^a^Pig population (thousands of heads) Eurostat data 2016, Switzerland Knoema, Serbian statistical office

### Percentage of pigs which are provided suitable enrichment materials

According to the respondents, 67% of pigs (median = 76%) across the 24 countries surveyed were provided with suitable enrichment materials. Within a country, between respondents, extensive variation was seen in the percentages regarding suitable enrichment materials (Table 1).

The percentage of suitable enrichment material was significantly different in TD-countries (Tail Docking countries) (mean 63% +/− sd 32.9%; median 70%) compared with No-TD countries (No Tail Docking Countries) (mean 89% +/− sd 14.8%; median 97%) (Z = 1.9, *P* = 0.048).

### Awareness on European and national recommendations and initiatives regarding prevention of tail biting and tail docking

Eighty-six percent (86%) of the veterinarians declared that they were aware of the Commission Recommendation regarding tail docking (EU 2016/336) [15] and 69% of the accompanying Commission Staff Working Document [16]. Fifty six percent (56%) replied they were aware of manuals, training opportunities or other supporting material developed and organised, mostly by the national governments, animal welfare councils/committees or research institutes.

Eighty-three percent (83%) reported the existence of a training for veterinary practitioners in No-TD countries, against 28% in TD-countries (*P* = 0.004). (see Table 2).

**Table 2:** Frequencies of answers in the tail docked (TD) and no routinely tail docked groups (No-TD)

	No-TD (respondents from countries with no Tail Docking)	TD (respondents from countries with Tail Docking)	*P*-value
Awareness of Commission Recommendations			
Yes	100%	84%	
No	0%	16%	0.22
Awareness of Commission Staff Working Document			
Yes	100%	65%	
No	0%	35%	0.08
Training for veterinary practitioners			
Yes	83%	28%	
No	17%	72%	**0.004**
Animal welfare advisory group			
Yes	88%	63%	
No	12%	37%	0.13
Role of veterinary practitioners			
Very important/Important	100%	66%	
Low involvement or importance	0%	34%	**0.07**
Policy for veterinary certificates			
Yes	38%	37%	
No	62%	63%	0.87
Recording of tail biting at the slaughterhouse			
Yes	100%	45%	
No	0%	55%	**0.003**
Effectivity of national initiatives			
Very effective/Effective	80%	3%	
Not very effective/not effective	20%	97%	**< 0.001**

*P*-value in bold means a *p*-value of < 0.05 which was considered significant

### National initiatives and the role of veterinary practitioners

Eighty (80%) of No-TD countries respondents thought national initiatives to reduce tail docking were “(very) effective” against only 3% in TD countries (*p* < 0.001).

The role of the veterinary practitioner was seen as (very) important by 100% participants from the No-TD group against 66% from the TD group (*P* = 0.07) (Table 2). Nobody replied that veterinarians have a deciding role.

### Recording of tail biting or tail docking at slaughterhouses

Recording of tail biting at the slaughterhouse was reported by 100% participants from the No-TD countries against 45% from the TD countries (*P* = 0.003) (Table 2).

### Most important practices suggested to prevent tail biting and to avoid routine tail docking of pigs

**Fig 1.**
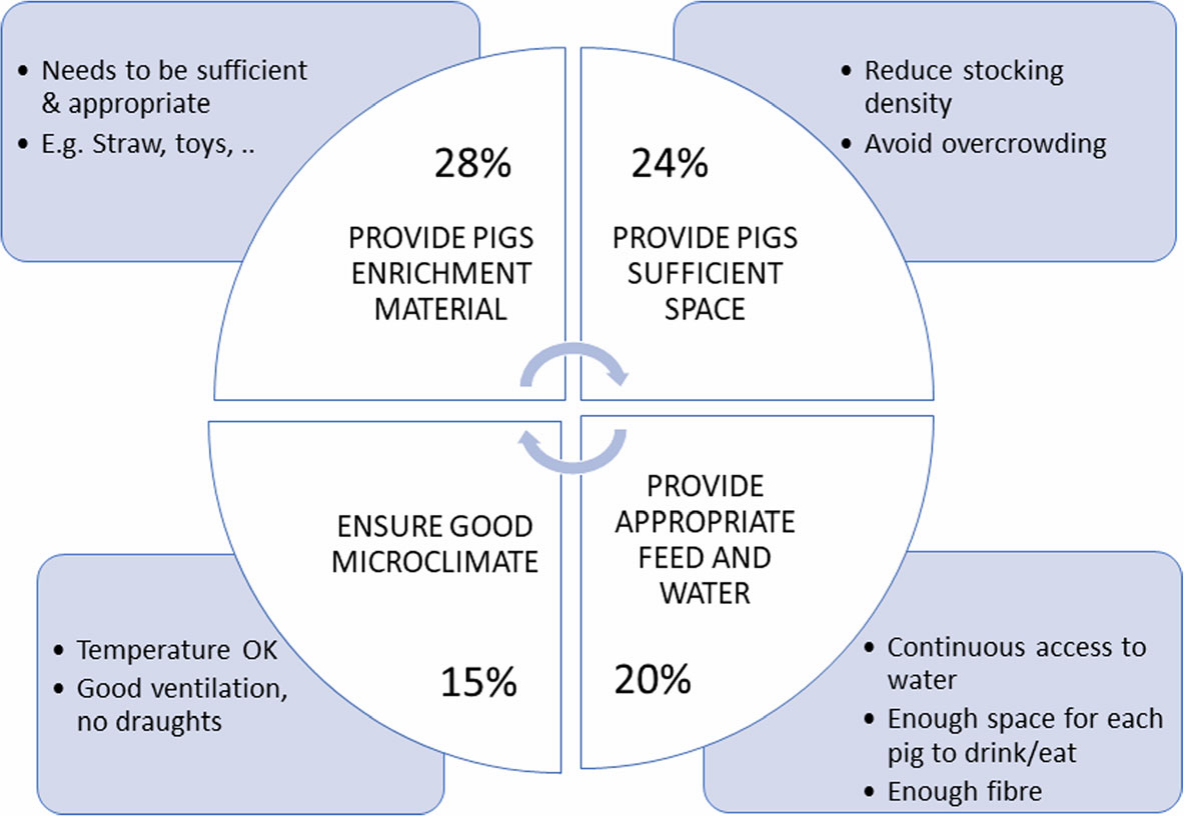
Most important practices suggested by veterinarians to prevent tail biting

The main practices suggested by veterinarians to prevent tail biting were: 1/ to provide sufficient and appropriate enrichment materials (quoted by 28%), 2/ stocking densities to be respected or reduced, i.e. avoid overcrowding (24%) 3/ appropriate feeding, watering and enough space for animals to drink and feed (20%) and 4/ an appropriate and reasonably stable microclimate (15%) (Fig. 1).

### The main challenges preventing veterinarians to advise to stop tail docking

**Fig 2.**
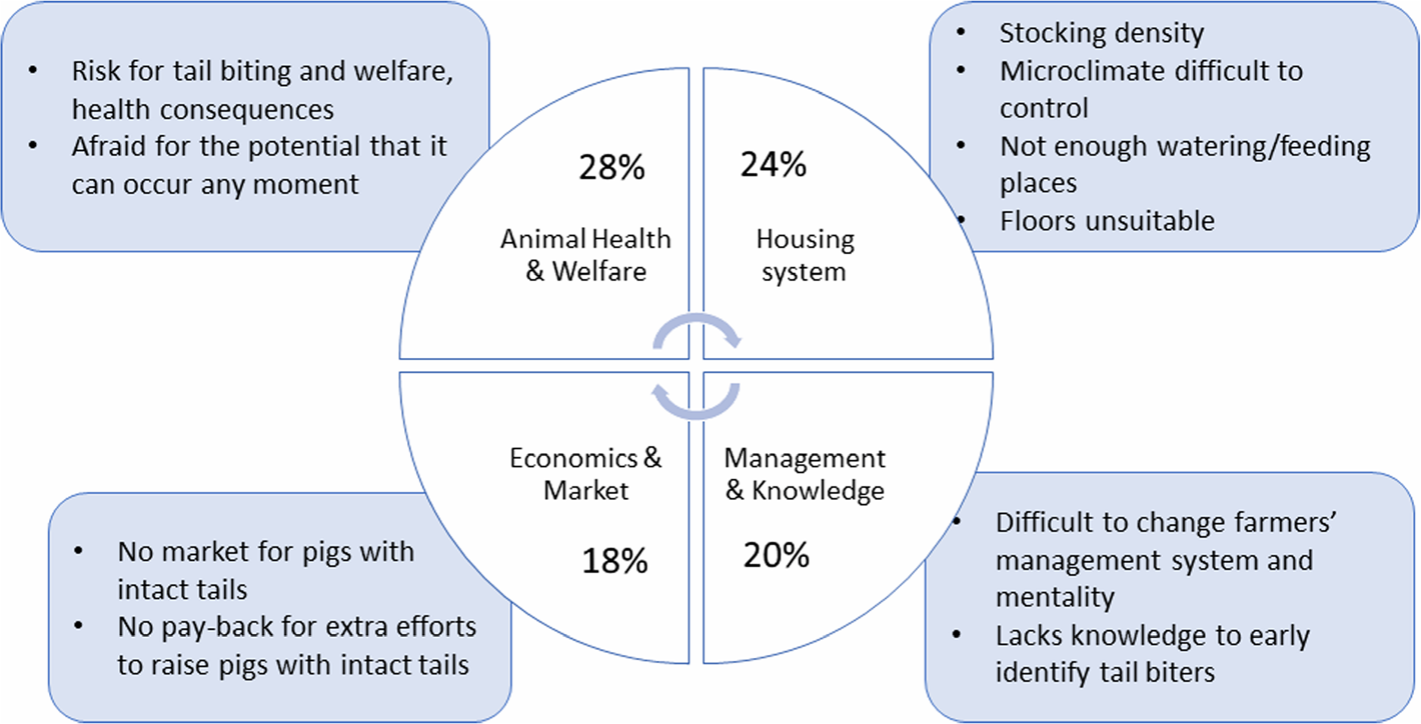
Main challenges to stop tail docking

The main challenges to stop tail docking quoted were: 1/ the risk for tail biting and the potentially welfare consequences (28%), 2/ inappropriate or insufficient housing (24%), 3/ related to farmers’ management, knowledge and (un)willingness or hesitation to change (20%) and 4/ related to economics and the market (18%) (Fig. 2).

### Top 3 benefits to farms not performing tail docking

The main benefits of not performing routine tail docking given were: 1/ improved animal welfare for the pigs, by avoiding the pain from the tail docking itself and the risk of consequent infections (46%), 2/ less labour and time input (15%), 3/ economic benefit for higher price with a label that requires intact tails and 4/ improved public image of pig farming (12%).

### Countries having stricter regulations on tail docking

Via desk-research, our study collected examples of EU and EFTA countries having stricter legislation than the EU Directive [1] (Table 3).

**Table 3:** Non exhaustive list of countries having stricter legislation then the EU Directive in relation to tail docking

Country	Legislation stricter than EU Directive
Denmark	Stricter legislation regarding rooting material, cooling, solid/drained floors and hospital pens, tail should be docked as little as possible (not more than ½ of the tail), if performed after the 4th day of life, piglets should be given long-lasting analgesia
Estonia	Veterinarian has to make the decision
Finland	Tail docking is forbidden since 2003, lower legal stocking densities
Germany	It is only allowed to tail-dock piglets up to an age of 4 days. If older, it has to be done by a veterinarian with anaesthesia (§5 TierSchutzGesetz). Since 2018, also stricter requirements apply regarding enrichment materials and a guidance to cut maximum 1/3 of the tail
Norway	Amputation of tails for medical reasons can only be performed by veterinarians Regulation for Housing of Swine of 2003, using anaesthesia and prolonged analgesia. As a consequence, it is not carried out any more Paragraph 10
Sweden	Tail docking is not allowed (SFS 1988:534 Paragraphs 2,4,10)
Switzerland	Removed from the list of mutilations that can be performed without anesthesia Animal Protection Ordinance, 2001

## Discussion

For most countries, reliable statistical data is not available on the amount of pigs with docked tails, tail biting lesions and how many pigs are provided with suitable and sufficient enrichment materials. The present survey relied upon a limited number of answers of experts in pig production from different countries despite reaching out extensively. This shows the need for more data collection on this topic. While the results presented in this document indicate the situation per country, it should be recognised that this might not reflect the situation in the whole of Europe, nor give a complete picture.

### Little progress in preventing tail docking

**Fig 3.**
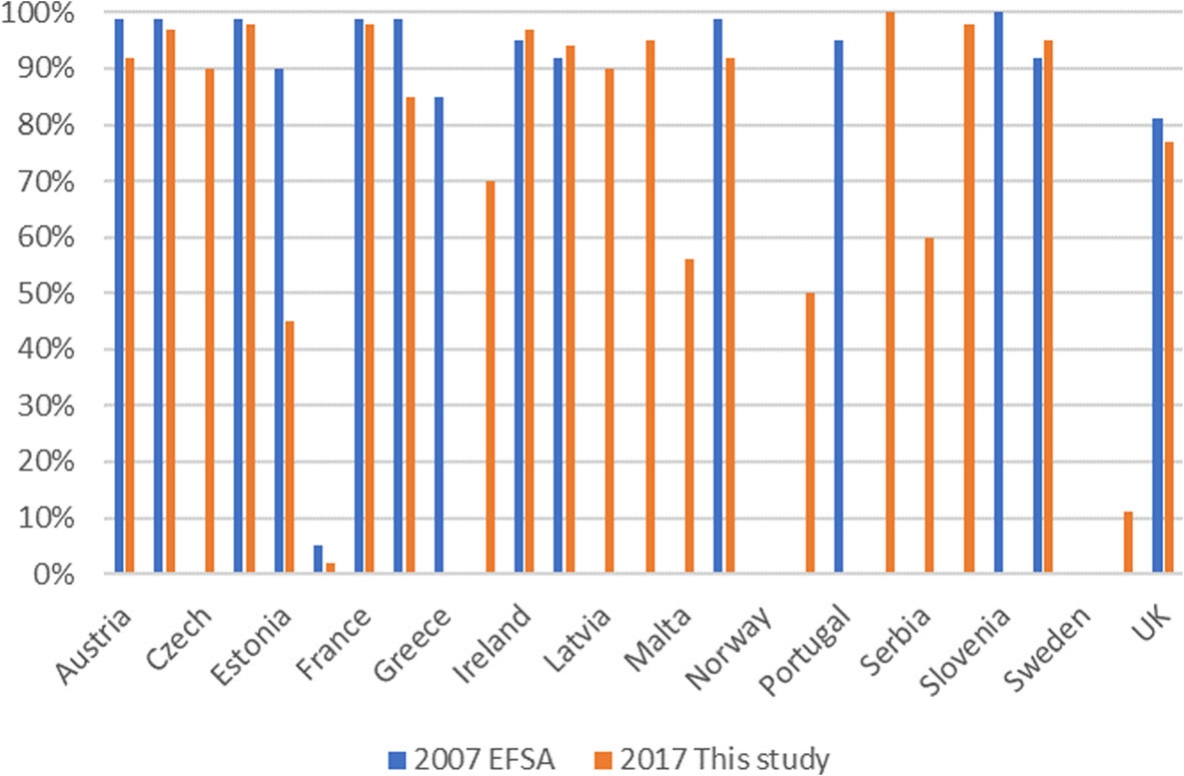
Percentage of docked pigs in 2007 (EFSA) and 2017 (our study)

Although routine tail docking is banned in the EU since 1994, the results of our survey show that little progress has been made in the last 20 years with still 77% (median = 95%) of the pigs being tail-docked in the 24 surveyed countries. Comparing with the percentages EFSA quoted in its 2007 report [2], the percentage of pigs being tail-docked has hardly decreased at all (see Fig. 3).

Four countries stand out, namely Finland, Norway, Sweden and Switzerland, as they stopped tail docking almost completely, without large problems with tail biting.

Most EU countries literally transcribed the EU pig directive text [1] in their national legislation. Exceptions to this are Denmark, Estonia, Finland, Norway, Sweden and Switzerland, which introduced stricter legislation (Table 3). Stricter implementation and control of the current legislation to enforce it properly, would be beneficial to pig welfare and also create a more level playing field for producers in different EU countries.

### Providing enrichment materials is vital to prevent tail biting, but not enough alone

While the legislation requires permanent access to a sufficient quantity of suitable enrichment and in details describes what is ‘suitable’ and ‘sufficient’, our survey results show that in reality knowledge and awareness is lacking. Only 67% of pigs (median = 76%) across the 24 EU countries surveyed were said to be given suitable enrichment materials. The association between providing suitable enrichment material and routine tail docking was significantly. The four countries that stopped tail docking replied to almost give all pigs sufficient and suitable enrichment material. Lack of proper enrichment material and need for appropriate space allowance were identified as the two main risk factors for tail biting. This expert opinion is in accordance with risk factors studies [2, 5, 6, 22, 23]. Several publications demonstrate that enrichment material and more specifically clean and fresh straw can substantially reduce the risk of tail biting [24]. Larsen [25] showed that a moderate amount of straw (150 g/pig/day) reduced the risk of injurious tail biting by more than two-fold, while docking reduced the risk by more than four-fold. Combining straw and increased space (1.2 m^2^ per pig) reduced the risk in undocked pigs to the same level as found in docked pigs kept under high stocking density (0.72 m^2^ per pig) without straw.

Tail docking has been reported as the most commonly applied management tool used to prevent tail biting. Hunter [26] recorded tail biting in 2.4% of docked and 8.5% of long-tailed pigs in the UK. In some cases, tail docking has reduced the prevalence of tail biting by up to 66% [27]. However, tail docking does not prevent tail biting completely. For example, sampling 18,500 docked pigs in French slaughterhouses in 2017, they found 62.6% of pigs having mild tail lesions and 2.21% with severe tail biting lesions [28].

Looking at the four countries that stopped tail docking almost completely, production systems are characterized by the use of enrichment materials (often straw), reduced stocking rates and high standards in relation to thermal comfort and air quality, health status, competition for food and space, and diet [20]. Both Sweden as Finland have a higher legally required space allowance than stipulated in the EU Directive [1]. A study interviewing Swedish pig farmers found that straw was used in 98% (61% straw only, 37% straw supplemented with other enrichment materials e.g. wood shavings) [24]. A Swiss study [29] showed that tail biting in undocked pigs can be reduced to similar levels as for docked pigs when the environment is suitably enriched.

Despite reporting high mean percentages of pigs having access to suitable enrichment material several countries still tail-docked more than 70% of pigs. This result may be explained through three main hypotheses: i) Due to the multifactorial nature of the problem [2, 6, 26], it may be difficult to predict and completely prevent episodes of tail biting [23]; ii) Another explanation is that of habit, i.e. the tendency to continue doing what has usually been done, and limited knowledge about the alternatives; iii) Yet another explanation is that raising pigs with intact tails may cost more, as a result of decreasing the stocking density, providing more enrichment material, and the labour costs involved including keeping a closer eye on any tendencies of tail biting.

### Measuring as precondition for improvement

Recording of tail biting at the slaughterhouse seems an important precondition for improvement. Recording was done in all countries which stopped routine tail docking against only 45% in the countries where tail-docking was common. Serious tail biting lesions should normally be recorded as part of the standard meat inspection system [30]. However, slaughterhouse data indicate that tail biting remarks from meat inspection data tend to severely underestimate on-farm prevalence of tail lesions [31, 32].

It is crucial to set up an effective monitoring program, both on farm as at the slaughterhouse, which records tail biting lesions and percentage of docked pigs. For monitoring and benchmarking purposes, both nationally as on a European scale, a grading scale should be developed to score tail docking (length of docking) and both old and new tail bite damage in a systematic, standardized way [32].

EUWelNet, a coordinated European Animal Welfare Network, has developed standardised training for EU inspectors in respect to provision enrichment materials, tail biting and tail docking [33].

### Preventing tail biting as part of herd health management

Seen the multi-factorial nature of tail biting [2, 6, 26], it needs a holistic health, welfare and biosecurity approach [14, 17]. While the deciding role to tail-dock or not, is up to the farmer, the farm veterinarian has an important role in doing a farm specific risk assessment and advising the farmer [10, 11]. Overall, 70% of the respondents see the role of vet practitioner as (very) important. Studies also showed that record keeping through an advisory service can help to lower the risk of tail biting, which is associated with improved farm performance [34, 35].

Some countries or farm assurance systems only allow farmers to tail-dock pigs after a veterinarian has issued a veterinary certificate or a veterinary statement [11]. However, most countries or veterinary practices do not have clear policies and guidelines when and after which assessment a certificate/statement can be issued. Every veterinary certificate issued should always follow the FVE 10 principles of certification [36]. These request amongst others that a veterinarian has to 1/only certify issues that he/she can ascertain 2/ avoid conflict of interest and 3/ does not allow economic or other pressures to compromise his/her impartiality.

Following the legislation, a veterinary certificate/ statement should only be given if the veterinarian has evidence of tail biting in the stable, when appropriate and sufficient enrichment material is present and after the farmer has tried to change e.g. housing, climate or stocking density.

### National action plans: Need for close collaboration between government, veterinary profession and pig industry

Our results show that countries that have stopped routine tail docking invest more in training for veterinary practitioners. The EU has requested all member countries to draw up an action plan to prevent tail docking by summer 2018. To make this plan work, it will be vital to work in close collaboration with the national pig veterinary practitioners and the pig industry in the country. Monitoring tail biting and tail docking and developing training materials and guidance should be part of the action plans. Several countries already developed support materials, such as Belgium [37], Denmark [38], Ireland [39], Spain [40] and the UK [41]. On a European level, materials were developed by FareWellDock [42] and the European Commission [43].

It is important to ensure that such useful materials reach all pig veterinarians and pig farmers in Europe.

## Conclusion

In the EU, routine tail docking is banned since 1994. Nevertheless, our study shows that the majority of pigs are still tail-docked. EU countries are developing national action plans to improve implementation and enforcement of the legislation. Our results show that it will be vital for countries to raise awareness and work out these plans in close collaboration with national pig veterinary practitioners and the pig industry. Private veterinarians in collaboration with all other actors have an important role to play in advising and supporting farmers. Monitoring tail biting and tail docking, both on farm as at the slaughterhouse, and developing training materials and guidance should be part of the above-mentioned plans. Veterinary certification can play a role, but should only be provided upon fulfilment of clearly specified criteria, based on proper investigations and clearly verifiable by the veterinarian and the authorities.

## Appendix

### Survey questions

***Open questions***

- Percentage of pigs tail-docked
- Percentage of pigs provided suitable enrichment materials
- List the top 3 practices suggested by veterinarians to prevent tail-biting and to avoid routine tail-docking of pigs
- List the main challenges to stop tail-docking
- List the main benefits for farmers to stop tail-docking
- Please share the best communication material you have seen to prevent tail-docking

***Dichotomous and multiple-choice questions***

- Do you have national legislation on tail docking in your country? (yes, no)
- Are you/your members aware of the Commission Recommendation laying down measures to reduce the need for tail-docking? (yes, no)
- Are you/your members aware of the Commission Staff Working Document on best practices to prevent routine tail-docking and on the provision of enrichment materials to pigs? (yes, no)
- Have you distributed these guidelines, or made them available to your members / colleagues? (yes, no)
- Has your national veterinary organisation or any other organisation organised training for veterinary practitioners on how they can assist operators to prevent tail biting pigs? (yes, no, I do not know)
- Is there any animal welfare advisory group (or similar) in the veterinary association(s) in your country? (yes, no, I do not know)
- How effective have these national initiatives been to reduce tail-docking in your opinion? (Very effective, effective, not very effective, not effective)
- Please describe the role of veterinary practitioners in such strategies on farms? (Very important, important, low involvement or importance)
- Does your association or practice have a policy on the issuing of veterinary certificates related to tail docking? (yes, no, I do not know)
- Are you aware if slaughterhouses in your country record the incidence of tail-docking and / or tail-biting (yes, no, I do not know)
- Is there a standard tail scoring methodology used? (yes, no, I do not know)
